# Magnetic Resonance Imaging of Radiation-Induced Brachial Plexopathy

**DOI:** 10.7759/cureus.60067

**Published:** 2024-05-10

**Authors:** Neha D Shetty, Rajasbala Dhande, Pratapsingh Parihar, Nikita Bora

**Affiliations:** 1 Radiodiagnosis, Datta Meghe Institute of Higher Education & Research, Wardha, IND; 2 Radiology, Datta Meghe Institute of Higher Education & Research, Wardha, IND

**Keywords:** radiation-induced brachial plexopathy, non traumatic plexopathy, brachial plexus injury, oncoradiology, palliative radiation therapy

## Abstract

This report illustrates the case of a 37-year-old woman following chemoradiotherapy for invasive ductal carcinoma of the right breast. The patient underwent surgery and received a radiation dose of 50 gray to the chest wall and 45 gray to the regional lymph nodes in 25 total fractions. She developed motor and sensory weakness in the right upper limb eight years after treatment. Brachial plexus neuropathy in cancer patients may result from either trauma to the plexus during surgery, the spread of cancer, or radiation therapy, and distinguishing between them may be difficult. The case highlights the importance of recognizing the signs, symptoms, and possible differential diagnosis of radiation-induced brachial plexopathy in cancer patients post-radiation therapy. It emphasizes the role of magnetic resonance imaging in the careful assessment and diagnosis of such a case.

## Introduction

Radiation-induced brachial plexopathy (RIBP) is an iatrogenic, progressively disabling aftereffect of adjuvant radiotherapy most commonly seen in breast cancer survivors. It is also encountered in patients with head and neck cancer, lymphoma, or lung carcinoma. Following breast cancer, the nerve injury is chronic and irreversible, occurring more commonly when axillary and/or supraclavicular nodes have been irradiated, as well as the breast and chest wall [1]. Clinically, RIBP is manifested initially by abnormal sensory symptoms, followed by motor weakness. The sensory manifestations include paresthesia, numbness, and dysesthesia (unpleasant sensations triggered by normal stimuli). The motor symptoms include weakness initially occurring in the thumb and progressing proximally up through the shoulder [2]. An analysis of research on breast cancer revealed that the risk of RIBP was less than 1% when fraction sizes ranged between 2.2 and 2.5 gray and the total dose did not exceed 40 gray [3]. In this report, we present the case of a 37-year-old woman with breast carcinoma who underwent surgery and chemoradiotherapy (CRT) and developed RIBP eight years after treatment.

## Case presentation

A 37-year-old woman presented with a painful lump rapidly increasing in size in her right breast. She underwent mammography, followed by a biopsy, which revealed invasive ductal carcinoma in her right breast. She was a habitual tobacco chewer. She was surgically treated with a modified radical mastectomy of the right breast, following which she underwent CRT. She went through 10 cycles of chemotherapy, which consisted of six cycles of the TCH regimen, which included carboplatin 900 mg, docetaxel 75 mg/m^2^, and trastuzumab 50 mg, followed by four cycles of trastuzumab alone. After four weeks of chemotherapy, radiotherapy was given using three-dimensional (3D) conformation radiation therapy with a total dose of 50 gray to the chest wall and 45 gray to the regional lymph nodes in 25 total fractions. It was delivered with six MV photons for 28 days. Post-CRT, she was advised to have regular yearly follow-ups for recurrence and spread of the tumor. Still, the patient was noncompliant, as she symptomatically did not have a recurrence of complaints.

Eight years post-CRT, the patient felt sensory defects in her right upper limb, which were initially characterized by numbness and tingling of the skin for the past year. The numbness and tingling were accompanied by minor pain. The patient also experienced motor weakness in the radial aspect of her right hand and was unable to flex her fingers or maintain a tight fist. Consequently, she was referred to a neurologist, where the neurological examination revealed an abnormal sensation on the radial side of the right hand, indicating findings consistent with a brachial plexus injury, suggesting a right C6 nerve root injury.

**Figure 1:**
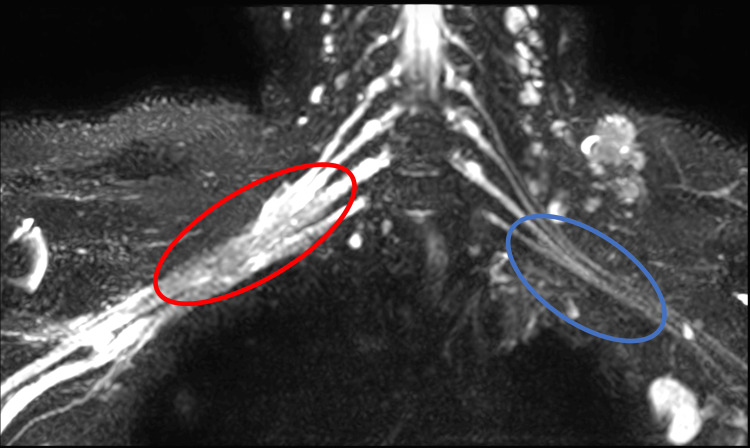
Three-dimensional magnetic resonance neurography The red circle indicates the thickened and edematous brachial plexus on the right, and the blue circle indicates the normal brachial plexus on the left.

**Figure 2:**
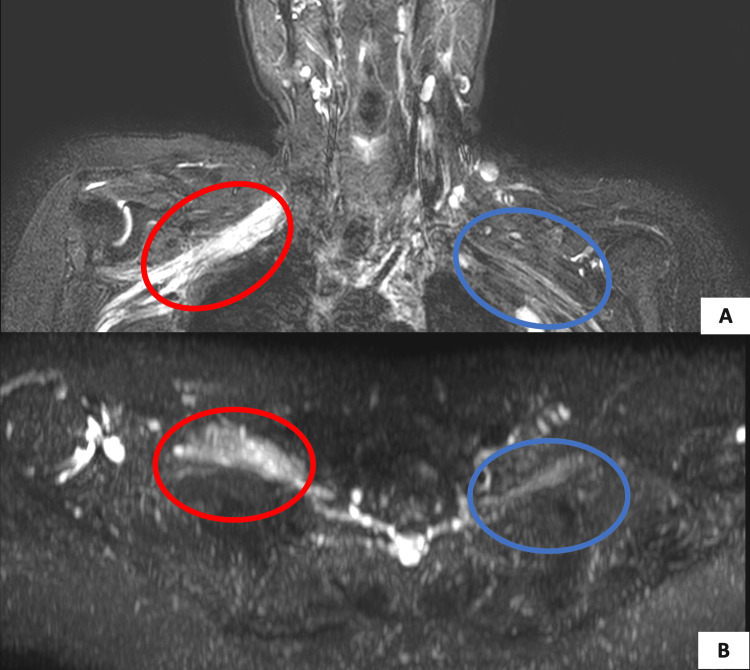
STIR sequence of MRI in (A) coronal and (B) axial showing the thickened brachial plexus on the right (red circle) compared to the left (blue circle) MRI, magnetic resonance imaging; STIR, short tau inversion recovery

**Figure 3:**
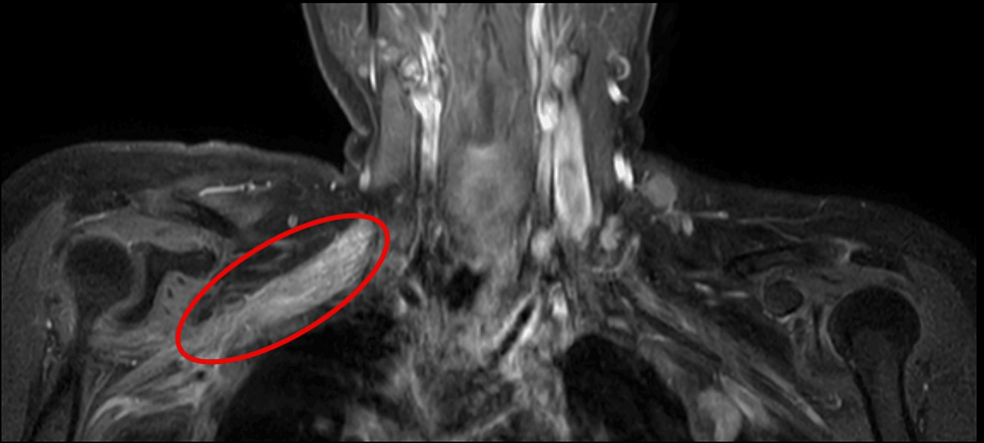
Contrast-enhanced T1W MRI image showing a uniformly enhancing brachial plexus on the right (red circle) MRI, magnetic resonance imaging; T1W, T1-weighted

The patient was thus advised to undergo cervical spine magnetic resonance imaging (MRI), which showed thickened, edematous cords and trunks of the right brachial plexus with focal high signal intensity in 3D magnetic resonance neurography (Figure 1) and short tau inversion recovery (Figure 2A, 2b) images. The 3D MR neurography depicts the origin of the brachial plexus from the cervical spine and its edematous and thickened nature on the right in comparison to the left. MR images post-contrast injection revealed uniformly enhancing cords of the brachial plexus on the right side (Figure 3). The absence of enhancing mass lesions in the MRI shows the lack of metastatic spread of the tumor, which is an important differential to be considered in cancer patients with branchial plexopathy. The cords of the branchial plexus are continuous, with no evidence of incoherence or gap in their course, which rules out surgical trauma to the brachial plexus.

Thus, the diagnosis of RIBP was derived from this patient. The patient was treated with B-isobutyl-G-aminobutyric acid (pregabalin) and duloxetine for the sensory symptoms of paresthesia. The motor symptoms were managed by physiotherapy, which focused on preserving the range of motion of the metacarpal joints of the hand and maintaining the power of the muscles.

## Discussion

Brachial plexopathy is a rare but important complication that causes disability in cancer patients. The causes of brachial plexopathy in cancer patients are tumor infiltration, radiation injury, and trauma to the plexus during surgery or anesthesia. Metastatic brachial plexopathy can present as major sensorimotor neurological deficits with accompanying pain or malignant lymphedema [4]. RIBP is nontraumatic damage to the brachial plexus that occurs delayed in onset, approximately four to five years, in cancer patients who have undergone radiation therapy. It occurs more frequently in patients treated for breast carcinoma and lymphoma. There has been a significant decrease in the frequency of acute brachial plexus injuries after radiation therapy as a direct result of advancements in radiation procedures. Nonetheless, with the growing acceptance of radiotherapy and conservative surgery as a treatment strategy, especially for early-stage breast cancer, the incidence of RIBP has also risen, and the reported incidence is 1.2% among them [5,6].

The pathophysiology of RIBP involves two phases of neuropathy following radiation. The first phase directly affects the electrophysiology and histochemistry of nerve fibers. A later phase involves the effects of irradiation immediately around the nerve and damage to the vascular supply of the nerves, causing fibrosis [7]. The fibrous tissue causes severe constriction of the nerve bundles. Furthermore, changes within the nerve bundles, such as wide-ranging loss of myelin, thickening of endoneurium and blood vessels, such as hyalinization, and obliteration of lumen, lead to microvascular insufficiency [8]. This ischemic demyelination thus causes a block in the conduction and propagation of action potential across nerve fibers. Hence, neuropathy is commonly observed in patients post-radiotherapy.

Brachial plexus neuropathy is directly associated with simultaneous surgical lymph node dissection, use of adjunct cytotoxic chemotherapy, accompanying vascular disease, and diabetes, along with factors such as radiation dosage, technique, and fractionation. The influences of the abovementioned elements contribute to the development of fibrosis surrounding the nerve bundles and within them [9]. According to Kori et al., only 19% of patients with RIBP complained of severe discomfort, compared to 80% of patients with tumor invasion of the brachial plexus [4]. Other symptoms of RIBP are erratic and may appear six months to 20 years following radiation treatment. The typical initial symptoms are motor weakness, lymphedema, paresthesia, and dysesthesia. In extreme situations, the shoulder joint’s range of motion may be restricted. Most patients experience a gradual course of symptoms. A third of patients show signs of fast deterioration and sensory impairments. In rare cases, the illness is mild, and its course may stop or reverse. RIPB and tumor-infiltrating brachial plexus disorders can present with motor weakness in the afflicted upper limb, but the symptoms occur fast and progress swiftly in tumor-infiltrating the brachial plexus [6].

MRI scans best distinguish RIBP from other brachial plexus disorders. Multiplanar imaging has considerable advantages for further outlining the pathology. In cases of tumor invasion into the brachial plexus, the lesion appears hyperintense, whereas radiation-induced fibrosis is iso- to hypointense on T2-weighted (T2W) images relative to muscle. Some cases of vascularized fibrous scar tissues can be hyperintense on T2W images, further complicating the diagnosis. Therefore, contrast-enhanced scans can be helpful in cases where enhancement is more striking in cases of tumor invasion into the brachial plexus in contrast to non-enhancing lesions noted in cases of fibrosis. A noninvasive method for differentiating a recurring tumor from radiation-induced fibrosis is MRI [9]. An accurate understanding of anatomical makeup and imaging-based identification of at-risk organs are essential for preventing RIBP [10].

Treatment plans for RIBP should emphasize improving function and reducing symptoms. One common and difficult symptom of RIBP is neuropathic pain, which is typically insufficiently relieved by traditional painkillers. Notable is the reported efficacy of a number of treatments, such as benzodiazepines, tricyclic antidepressants, antiepileptic agents, and non-opioid analgesics [11]. When there is progressive motor weakness and the discomfort is not improving with conservative treatments, surgery is recommended.

## Conclusions

The complex presentation and constrained management options of RIBP are challenging problems. Especially for a patient already dealing with the diagnosis of cancer, the physical and mental strain of having to deal with RIBP can be demanding. Reducing the patient’s pain is the aim of treatment. Accurate diagnosis and its distinction from its differentials must be prompt. The most crucial thing is to distinguish it from brachial plexopathy brought on by tumors. This side effect should be known to the radiation oncologist, and following radiation therapy, patients should have routine counseling and clinical assessments conducted. The goal of management should be to enhance function and reduce symptoms in order to improve quality of life.
